# COPD prescription patterns after the 2018 Japan floods: analysis of national data

**DOI:** 10.1038/s41598-026-55662-y

**Published:** 2026-06-01

**Authors:** Tatsuki Takahashi, Shuhei Yoshida, Hiroshi Iwamoto, Yasushi Horimasu, Shinichiro Ohshimo, Noboru Hattori, Masatoshi Matsumoto

**Affiliations:** 1 Department of Molecular and Internal Medicine, Graduate School of Biomedical and Health Sciences, Hiroshima University, 1-2-3 Kasumi, Minami-ku, Hiroshima, 734-8551 Japan; 2 Department of Community-Based Medical Systems, Graduate School of Biomedical and Health Sciences, Hiroshima University, Hiroshima, Japan; 3 Department of General Internal Medicine, Hiroshima University Hospital, Hiroshima, Japan; 4 Department of Emergency and Critical Care Medicine, Graduate School of Biomedical and Health Sciences, Hiroshima University, Hiroshima, Japan

**Keywords:** Natural disasters, Climate change, Global warming, Epidemiology, COPD exacerbation, Flood disasters, Diseases, Health care, Medical research, Risk factors

## Abstract

Climate change has led to an increase in flood disasters worldwide, raising concerns about their potential impact on respiratory health. This study investigated the impact of the 2018 Japan Floods, one of the largest flood disasters in Japan’s history, on prescription rates for chronic obstructive pulmonary disease (COPD). To examine the impact of the disaster on COPD-related prescriptions, we analysed anonymised health insurance claims data from the Japanese National Database. The study focused on residents of three prefectures that included the most severely affected areas during the 2018 Japan Floods, covering a 2-year observation period—1 year before and 1 year after the disaster. First, we compared new prescriptions of long-acting inhaled COPD medications after the disaster between victims and non-victims among individuals who had not received such prescriptions during the previous year (n = 3,674,535). Second, we examined prescriptions for antibiotics or systemic steroids after the disaster among individuals already receiving long-acting inhaled COPD medications before the disaster (n = 18,059), as indicators of COPD exacerbations. Cox proportional hazards models showed that new prescriptions for long-acting inhaled COPD medications were significantly higher among disaster victims than among non-victims in the year following the 2018 Japan Floods (aHR: 1.56; 95% CI [1.25–1.95]). Difference-in-differences analysis showed that exacerbation-related prescriptions significantly increased among disaster victims compared with non-victims (aROR: 1.50; 95% CI [1.06–2.11]). New prescriptions for COPD controller medications and exacerbation-related prescriptions significantly increased in populations affected by the 2018 Japan Floods.

**Supplementary Information:**

The online version contains supplementary material available at 10.1038/s41598-026-55662-y.

## Introduction

Global climate change has increased the frequency and intensity of heavy rainfall and flooding, which may contribute to adverse respiratory health outcomes^1,2^. Flood victims face exposure to various hazardous substances, both acutely and over extended periods^3–5^, which may adversely affect chronic obstructive pulmonary disease (COPD). COPD is a prevalent respiratory disorder associated with substantial morbidity and healthcare impact worldwide^6^. In addition to smoking, exposure to biomass, occupational irritants, and air pollution has been reported as a contributing factor in the development of COPD^7–11^. COPD exacerbations are triggered by infection, air pollution, or unknown causes and are associated with disease progression and poor prognosis^12–14^. Understanding potential risk factors for COPD, including environmental exposures, is important for timely treatment and prevention of disease progression. However, little is known about the impact of flood disasters on COPD. Given the rapid increase in flood disasters globally, understanding the impact of flood disasters on COPD is critical for both clinical management and public health.

Heavy rainfall struck western Japan between June 28 and July 8, 2018, causing widespread landslides and river flooding. The 2018 Japan Floods, one of the largest flood disasters in Japan’s recorded history, resulted in 263 fatalities, 484 injuries, and 8 missing persons. In addition, 29,473 houses were affected, including those completely destroyed, half-destroyed, partially destroyed, or flooded above the floor level.

The aim of this study was to investigate the impact of flood disasters on the prescription rates of COPD medications. We used a large-scale public health insurance database to compare new prescriptions for COPD controller medications and exacerbation-related prescriptions between victims and non-victims of the disaster within 1 year after the 2018 Japan Floods.

## Methods

This retrospective cohort study analysed data from the Japanese National Database of Health Insurance Claims (NDB), an administrative claims database in Japan. In Japan, all citizens must enrol in public health insurance, and the NDB contains comprehensive records of all medical services provided. Owing to the anonymised nature of the NDB data, the requirement for informed consent was waived by the Ethics Committee for Epidemiological Research at Hiroshima University. This study was approved by the Ethics Committee for Epidemiological Research at Hiroshima University (approval number E-1688), and permission for data use was granted by the Ministry of Health, Labour and Welfare (authorization number 1223–2). All methods were performed in accordance with the relevant guidelines and regulations and with the Declaration of Helsinki.

### Setting

From June 28 to July 8, 2018, western Japan was struck by torrential rainfall, resulting in extensive landslides and river flooding. We analysed NDB data from residents of Hiroshima, Okayama, and Ehime prefectures, which included the regions most severely affected by the 2018 Japan Floods and accounted for 90% of all fatalities from the disaster. The observation period was between July 2017 and June 2019, covering 1 year before and after the 2018 Japan Floods. Individuals aged 40 years or older were included in this study, and data extracted from the NDB included information on sex, age group, and names and quantities of prescribed medications. COPD prevalence markedly increases in middle-aged and older adults, and epidemiological studies commonly evaluate COPD in populations aged ≥ 40 years^6^. Therefore, this study focused on individuals aged 40 years or older to improve the clinical interpretability of COPD-related prescription patterns in this claims database analysis.

Individuals directly affected by the 2018 Japan Floods are registered as disaster victims in the NDB. The criteria for qualification as disaster victims are as follows:

- The individual’s residence was completely destroyed, partially destroyed, flooded above floor level, or presented similar damage.
- The individual experiences a loss of economic support because of a family member’s death, injury, disappearance, unemployment, cessation of business operations, or loss of income due to the disaster.

### Outcomes

**Fig. 1:**
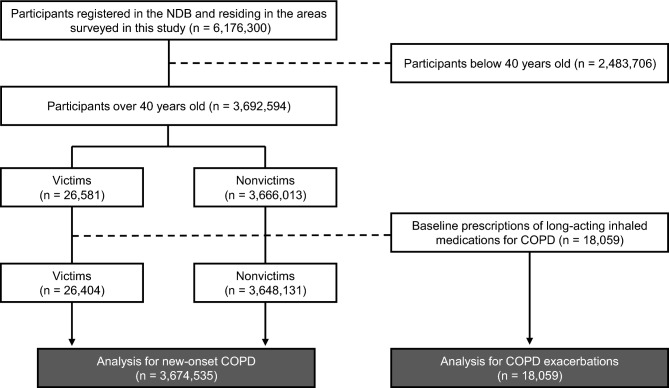
Study flow. Among those registered with the NDB, 3,692,594 individuals who met the study criteria were included. COPD, Chronic obstructive pulmonary disease; NDB, National health insurance claims database.

First, we compared new prescriptions of long-acting inhaled medications that were indicated solely for COPD between disaster victims and non-victims among individuals without such prescriptions during the previous year (n = 3,674,535) (Fig. 1). Second, we investigated prescription rates of oral corticosteroids or antibiotics among pre-existing COPD patients (those on long-acting inhaled medications before the disaster), as indicators of exacerbations (Fig. 1). Both outcomes were compared between disaster victims and non-victims.

Long-acting inhaled COPD medications and the drugs prescribed for exacerbations included in the analysis are listed in Tables S1 and S2 in the Supporting Information. In this study we defined long-acting inhaled COPD medications as those indicated exclusively for COPD treatment. These medications, along with their potential substitutes, are not available over the counter in Japan. Therefore, the data used in this study comprehensively covered the medications used for COPD treatment in the study population.

### Statistical analysis

Logistic regression analysis was used to compare the baseline prescription rates of long-acting inhaled COPD medications during the year before the disaster between disaster victims and non-victims.

For new prescriptions of COPD controller medications, cumulative hazard functions were estimated for the victim and non-victim groups using the Kaplan–Meier method. Cox regression analysis was performed using a new prescription for long-acting inhaled COPD medications as an event of interest. The follow-up ended either at the time of prescription or at the end of the observation period. Non-victims were the reference group, and age and sex were explanatory variables.

As a sensitivity analysis, we additionally performed a difference-in-differences analysis for prescriptions of long-acting inhaled COPD medications to evaluate the robustness of the main findings. July 2017 (1 year pre-disaster) was used as the baseline. Adjusted ratios of odds ratio (aRORs) and their 95% confidence intervals (CI) were calculated for each month after July 2017. The monthly aRORs represent how the odds ratios changed relative to July 2017. The difference-in-difference analysis, a quasi-experimental method, is used to compare the changes in event rates after the disaster while accounting for pre-disaster trends between victim and non-victim groups. Theoretically, this method allows comparisons between both groups without being influenced by baseline health disparities, excluding the effects of potential confounding factors. A linear probability model with an interaction term for disaster status (victims and non-victims) was used for the difference-in-difference analysis. Robust standard errors clustered by individuals were applied to account for repeated measurements in the same individual. The estimates and their 95% CIs for the baseline and comparison periods were reported. To assess the temporal trend of exacerbation-related prescriptions following the disaster, we conducted a month-by-month difference-in-differences analysis covering the 12 months before and after the flood event.

For exacerbation-related prescriptions, the difference-in-differences approach was used to assess the rate of outcome occurrence between disaster victims and non-victims who had been using long-acting inhaled COPD medications before the disaster.

Age- and sex-stratified subgroup analyses were conducted, including stratification by age group (< 65 and ≥ 65 years), to examine differences in prescription patterns between disaster victims and non-victims. To further explore heterogeneity among older individuals, we conducted an additional age-stratified analysis dividing participants aged ≥ 65 years into two groups (65–79 and ≥ 80 years).

All analyses were conducted using STATA/MP Parallel Edition version 16.1 (StataCorp, 2019, College Station, TX, USA). Statistical significance was defined as a two-sided p-value of < 0.05.

## Results

### Baseline characteristics

Overall, 6,176,300 individuals in the NDB dataset were enrolled. Of these, 3,692,594 individuals aged ≥ 40 years were included in the analysis (Fig. 1). The proportion of older individuals aged ≥ 65 years was higher among disaster victims than among non-victims (61.68% v 48.99%; Table 1). Additional age distribution across finer age groups is presented in Table S3 in the Supporting Information. One hundred and seventy-seven victims and 17,882 non-victims were prescribed long-acting inhaled COPD medications during the year before the disaster (Table 1). In the logistic regression analysis, the prescription rate of long-acting inhaled COPD medications during the year before the disaster did not significantly differ between disaster victims and non-victims (Table S4 in the Supporting Information).

**Table 1:** Participants’ characteristics.

	Victims (n = 26,581)	Non-victims (n = 3,666,013)
Age group, n (%)		
40–64 years	10,185 (38.32)	1,870,036 (51.01)
≥ 65 years	16,396 (61.68)	1,795,977 (48.99)
Sex, n (%)		
Male	11,591 (43.61)	1,679,186 (45.80)
Female	14,990 (56.39)	1,986,827 (54.20)
Baseline (pre-disaster) prescriptions of COPD long-acting inhaled medications, n (%)		
All people	177 (0.67)	17,882 (0.49)
40–64 years	18 (0.18)	2038 (0.11)
≥ 65 years	159 (0.97)	15,844 (0.88)
Male	150 (1.29)	15,039 (0.90)
Female	27 (0.18)	2843 (0.14)

COPD, Chronic obstructive pulmonary disease.

### Increase in new prescriptions for COPD controller medications among disaster victims

**Fig. 2:**
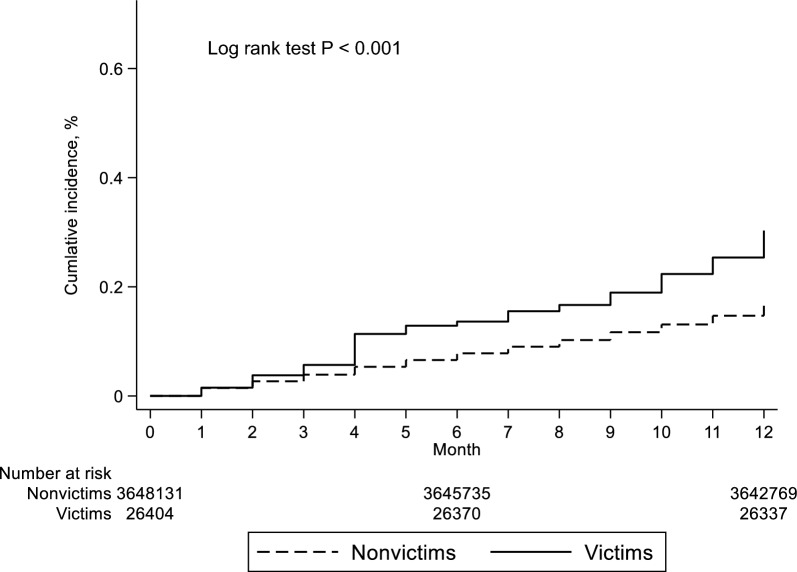
Cumulative rate of long-acting inhaled medications for COPD. Kaplan–Meier curves showed that victims had a significant increase in new prescriptions of long-acting inhaled medications for COPD over time compared with non-victims. COPD, Chronic obstructive pulmonary disease.

Disaster victims had higher new prescription rates than non-victims (Table S5 in the Supporting Information). The Cox proportional hazards model revealed that disaster victims had a significantly higher rate of new prescriptions for long-acting inhaled COPD medications than non-victims (aHR: 1.56; 95% CI [1.25–1.95], *p* < 0.001; Table 2). Figure 2 shows the cumulative incidence rates of new prescriptions for long-acting inhaled COPD medications for disaster victims and non-victims. The log-rank test indicated that victims had significantly higher rates of new prescriptions than non-victims (*p* < 0.001). In a sensitivity analysis using a difference-in-difference approach, prescriptions of long-acting inhaled COPD medications were also significantly higher among disaster victims than among non-victims (Table S6 in the Supporting Information).

**Table 2:** Comparison between multivariate Cox proportional hazards models for new prescription of long-acting inhaled medications for COPD for disaster victims v non-victims (control).

	Adjusted hazard ratio [95% CI]
All participants	1.56 [1.25–1.95]
Age group	
40–64 years	2.02 [1.17–3.48]
≥ 65 years	1.49 [1.17–1.90]
Sex	
Male	1.66 [1.31–2.10]
Female	1.13 [0.62–2.05]

CI, Confidence interval; COPD, Chronic obstructive pulmonary disease.

**Fig. 3:**
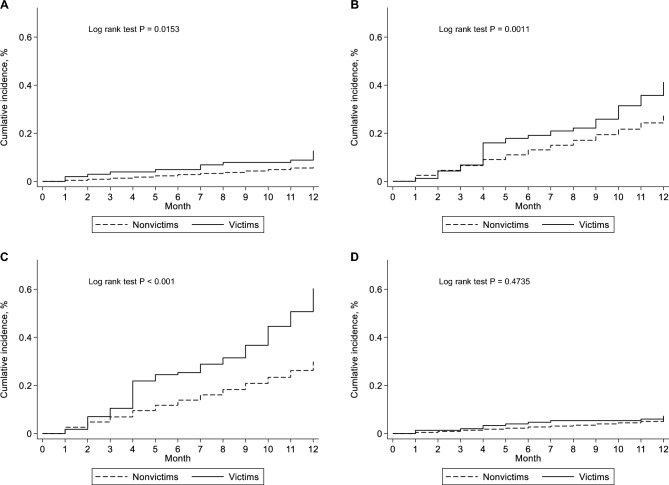
Subgroup analysis of prescription for long-acting inhaled medications for COPD. (**A**) 40–64 years old, (**B**) ≥ 65 years old, (**C**) Male, (**D**) Female. COPD, Chronic obstructive pulmonary disease.

The subgroup analysis of new prescriptions of long-acting inhaled COPD medications is shown in Fig. 3. In the age-specific analysis, an increase in new prescriptions for COPD controller medications was observed from 1 month after the disaster among individuals under 65 years, whereas a delayed increase was observed from November (4 months after the disaster) among those aged 65 years and older (Fig. 3). In the sex-specific subgroup analysis, male victims had a significantly higher rate of new prescriptions than male non-victims (aHR 1.66; 95% CI [1.31–2.10]) (Fig. 3, Table 2). There was no significant difference in the new prescription rate between female victims and female non-victims.

### Increased prescriptions for COPD exacerbation among disaster victims

**Fig. 4:**
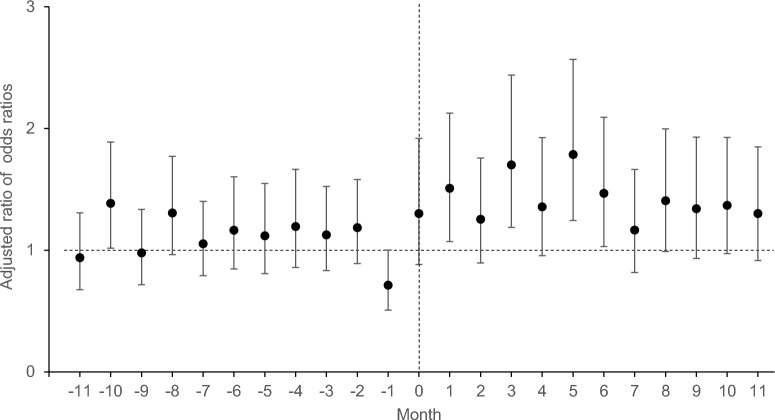
Monthly time-course of adjusted ratios of odds ratios for prescription rates of exacerbation-related medications among disaster victims versus non-victims. Prescription rate was compared in individuals who had a baseline prescription of long-acting inhaled medications for COPD and evaluated using a difference-in-differences analysis (n = 17,756). Month 0 was July 2018 when the floods occurred. The horizontal dashed line represents an adjusted ratio of odds ratios of 1. COPD, Chronic obstructive pulmonary disease.

Figure 4 shows the monthly time-course of aRORs for prescription rates for COPD exacerbation between disaster victims and non-victims. Prescription rates for COPD exacerbations among victims increased significantly compared with non-victims (aROR: 1.50, 95% CI [1.06–2.11]) in the month following the disaster (August 2018). The increased trend in exacerbation-related prescriptions of victims persisted for 6 months, during autumn and winter (October 2018 and December 2018) with aRORs [95% CI] of 1.64 [1.15–2.35] and 1.72 [1.20–2.46], respectively.

**Fig. 5:**
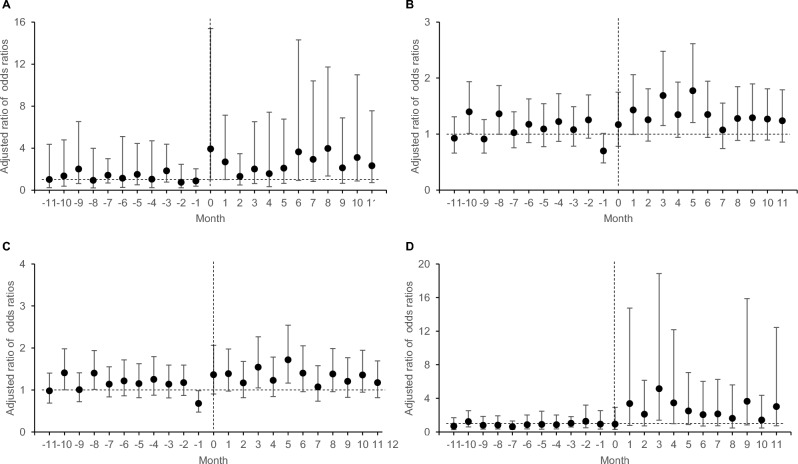
Monthly time-course of adjusted ratios of odds ratios for prescriptions of exacerbation-related medications in subgroup analyses comparing disaster victims and non-victims. (**A**) 40–64 years old, (**B**) ≥ 65 years old, (**C**) Male, (**D**) Female. Month 0 was July 2018 when the floods occurred. The horizontal dashed line represents the adjusted ratio of odds ratios of 1.

In the age-stratified subgroup analysis of exacerbation-related prescriptions, disaster victims under 65 years showed significant increases in the month of the disaster with aRORs [95% CI] of 3.92 [1.14–13.44] (Fig. 5). Among disaster victims aged 65 years and older, significant increases were observed at 3 and 5 months after the disaster, with aRORs [95% CI] of 1.69 [1.15–2.48] and 1.78 [1.21–2.61], respectively (Fig. 5). In the sex-specific subgroup analysis, both men and women disaster victims showed a significant increase in prescriptions for COPD exacerbation treatments at 3 months post-disaster, and there was also increased prescription at 5 months in men (Fig. 5).

In an additional analysis further stratifying individuals aged ≥ 65 years, new prescriptions of long-acting inhaled COPD medications were significantly increased among those aged 65–79 years, whereas no significant increase was observed among those aged ≥ 80 years (Fig. S1 in the Supporting Information). Exacerbation-related prescriptions showed no significant increase among those aged 65–79 years but were significantly increased among individuals aged ≥ 80 years (Fig. S2 in the Supporting Information).

## Discussion

In this study, we demonstrated that new prescriptions for long-acting inhaled COPD medications significantly increased among disaster victims compared with that among non-victims following the 2018 Japan Floods. Additionally, we observed an increased prescription rate for COPD exacerbations that began shortly after the disaster, which persisted for 6 months following the disaster. These findings indicate that disaster victims had higher rates of COPD-related prescriptions than non-victims following the 2018 Japan Floods, which may suggest an association between flood disasters and increased COPD-related treatment needs.

This study demonstrated an increase in the initiation of long-acting inhaled medications, which are indicated exclusively for patients with a diagnosis of COPD, among flood victims. Two possible explanations may account for this finding. First, flood-related exposures may have contributed to the emergence or recognition of COPD requiring treatment. Second, individuals with pre-existing but untreated COPD may have experienced worsening respiratory symptoms after the disaster, prompting treatment initiation. Floods can generate airborne particulate matter containing toxic chemicals, heavy metals, and microbial endotoxins from resuspended sediments. These pollutants may persist in the environment long after floodwaters recede, leading to sustained respiratory exposure^4,5^. Cleaning activities and the use of disinfectants are associated with an increased risk of COPD^15,16^, and flood victims can be exposed to various pollutants associated with cleaning activities^3^. Damage to homes may lead to prolonged exposure to pollutants and moulds, while displacement to temporary housing, the loss of family members, and financial hardship can cause psychological distress; both factors may be associated with respiratory symptoms associated with COPD after a delay following the disaster^5,17^. In the present study, subgroup analysis showed that new prescriptions of COPD controller medications increased from the month of the disaster among disaster victims under 65 years, while the increase in those aged 65 and older began in November, 4 months post-disaster. We could speculate that acute exposures, such as pollutants or cleanup-related irritants may have had greater influence on younger individuals, whereas prolonged impacts of the flood disaster may be associated with delayed increase of COPD controller prescriptions in older adults. The present results suggest that short- and long-term effects of flood disasters may be associated with respiratory damage and symptom worsening, leading to increased prescriptions of COPD controller medications.

In this study, exacerbation-related prescriptions significantly increased among disaster victims compared with that among non-victims in the month following the disaster, and this trend persisted during the winter months until 6 months post-disaster. Exposure to air pollutants and moulds is a risk factor for COPD exacerbation^18,19^, and inhaling fine particulate matter dispersed from sediment and debris caused by floods may have been associated with the observed increase in exacerbations. Oluyomi et al. reported increased upper respiratory symptoms 12 months after Hurricane Harvey in individuals who had contact with debris, sewage, or visible mould after floods, suggesting a prolonged influence of flood disasters^3^. Moreover, airborne pollutants can increase susceptibility to respiratory infections, leading to COPD exacerbations by impairing respiratory immune defences through alterations of antiviral mechanisms, reducing mucociliary clearance, and increasing airway inflammation^20–22^. In the present study, age-stratified analysis of COPD exacerbation prescriptions showed a significant increase among disaster victims under 65 years during the flood month and one month after, while in those aged 65 and older, the increase was evident 3 and 5 months post-disaster in the autumn and winter periods. It can be speculated that younger individuals were more affected by acute exposures. Younger disaster victims may have been more likely to participate in recovery or cleanup activities after the flood, potentially resulting in greater exposure to respiratory irritants such as dust, debris, and mould^23^. In contrast, among older adults, delayed flood-related effects or seasonal factors such as infections may have been associated with increased exacerbation-related prescriptions in flood victims. The present findings suggest that both the immediate and long-term impacts of the flood disaster may be associated with an increased risk of COPD exacerbations.

In the sex-specific subgroup analysis, new prescription for long-acting inhaled COPD medications increased only among male victims compared to non-victims, a pattern that contrasts with the findings for exacerbation-related prescriptions, which increased among both male and female victims. One possible explanation for this discrepancy is that underdiagnosis of new-onset COPD in women following flood disasters may have contributed to the observed findings. A delay between the onset and diagnosis of COPD is common, and women are more likely to experience delayed diagnosis than men, and this may have contributed to the lack of observed increase in new prescriptions among females^24,25^. Although this interpretation remains speculative, it may suggest the need for clinical vigilance regarding new-onset COPD in both men and women following future flood disasters.

## Limitations

This study had some limitations. First, COPD controller medications analysed in this study were limited to long-acting inhalers specifically indicated for COPD. Consequently, patients with mild COPD using only short-acting inhalers and those prescribed inhalers indicated for bronchial asthma were excluded from the analysis. Second, the comparison of new prescriptions for COPD controller medications between disaster victims and non-victims was adjusted only for age and sex. However, the prescription rates for these medications during the year before the disaster did not differ between the two groups. This pre-disaster parity suggests that unmeasured baseline factors—such as smoking status, socioeconomic status, or preexisting respiratory diseases—were unlikely to have substantially biased the findings. Moreover, we employed a difference-in-differences analysis to assess exacerbation-related prescriptions, which helps mitigate the influence of time-invariant unmeasured confounding. However, residual confounding due to unmeasured factors such as smoking status and socioeconomic status cannot be excluded. Third, COPD exacerbations were defined based on prescription data (systemic corticosteroids or antibiotics), which may have resulted in misclassification and may not fully reflect the actual clinical condition of patients. This proxy definition should be interpreted with caution as it may not fully capture clinical exacerbations. Fourth, it is worth considering that the greater accessibility of prescriptions for existing and new patients might be associated with medical expense support provided to disaster victims, including the elimination of out-of-pocket expenses through disaster victim certification. However, within Japan’s healthcare system, the rise in medical care demand due to out-of-pocket expense exemptions is considered minimal. In Japan, drug prices are standardised nationwide at lower rates than in many other countries^26^. Moreover, co-payment rates are as low as 30% at most. Therefore, the decline in medication usage in response to rising outpatient costs is generally small, with a price elasticity ranging from − 0.125 to − 0.076^27^. Accordingly, out-of-pocket costs are not a major obstacle to seeking medical care, and the impact of treatment cost exemptions on the increase in prescriptions is likely minimal. Fifth, information on deaths during the follow-up period was not available in this database; therefore, we were unable to account for mortality as a competing event in the analysis. Finally, we were unable to follow individuals who moved out of Hiroshima, Okayama, and Ehime prefectures during the study period. However, only approximately 1.6% of the total population relocated outside the study area in 2019, suggesting that the effect on our results is likely negligible. The total population in the study region was about 6.07 million (6,074,466 in 2018), and our database captured roughly 94% of this population. This suggests that most residents visited a healthcare facility at least once over the 2-year study period. Japan’s universal health insurance system, characterized by low drug prices and co-payments, as well as unrestricted access to medical services, likely facilitated this high utilisation. While some limitations exist, the study’s use of a large-scale national database, patient-level evaluation, year-long post-disaster follow-up, and multivariable and difference-in-differences analyses provide useful insights into COPD-related prescription patterns following flood disasters.

## Conclusions

In conclusion, this longitudinal study demonstrates a significant increase in both new COPD controller medication prescriptions and exacerbation-related treatments among individuals affected by the 2018 Japan Floods. These findings suggest a possible association between flood disasters and increased COPD-related prescriptions, which may warrant increased clinical attention to COPD in post-disaster settings.

## Supplementary Information

Supplementary Information.

## Data Availability

The datasets used during our study are not publicly available because of the rule decided by the Ministry of Health, Labor and Welfare in Japan. Data are available for researchers with permission from the Ministry of Health, Labor and Welfare (https://www.mhlw.go.jp/stf/seisakunitsuite/bunya/kenkou_iryou/iryouhoken/reseputo/index.html).

## Abbreviations

CI: Confidence interval
COPD: Chronic obstructive pulmonary disease
NDB: National health insurance claims database
