# Supplementary material for: COPD prescription patterns after the 2018 Japan floods: analysis of national data
Source: Sci Rep. 2026 Jun 1;16:24858. doi: 10.1038/s41598-026-55662-y (PMC13458695; doi:10.1038/s41598-026-55662-y)
Supplement: Supplementary file 1 [file 41598_2026_55662_MOESM1_ESM.docx]

**Table S1 List of long-acting inhaled medications for chronic obstructive pulmonary disease**

| **Category** | **Drugs** |
| --- | --- |
| Long-acting β-agonists (LABA) | Formoterol fumarate hydrate |
|  | Indacaterol maleate |
| Long-acting muscarinic antagonist (LAMA) | Aclidinium bromide |
|  | Umeclidinium bromide |
|  | Glycopyrronium bromide |
|  | Tiotropium bromide hydrate |
| LAMA/LABA | Umeclidinium bromide, vilanterol trifenatate |
|  | Glycopyrronium bromide, indacaterol maleate |
|  | Tiotropium bromide hydrate, olodaterol hydrochloride |
|  | Glycopyrronium bromide, formoterol fumarate hydrate |
| Inhaled corticosteroid (ICS)/LAMA/LABA | Fluticasone furoate, umeclidinium bromide, vilanterol trifenatate |
|  | Budesonide, glycopyrronium bromide, formoterol fumarate hydrate |

**Table S2 List of medications for exacerbations of chronic obstructive pulmonary disease**

| Category |  |  | Category |  |
| --- | --- | --- | --- | --- |
| Penicillin | Benzylpenicillin potassium |  | Monobactam | Aztreonam |
|  | Benzylpenicillin benzathine hydrate |  | Penem | Faropenem |
|  | Ampicillin |  | Glycopeptide | Vancomycin hydrochloride |
|  | Bacampicillin hydrochloride |  |  | Teicoplanin |
|  | Amoxicillin hydrate |  | Lipopeptide | Daptomycin |
|  | Sultamicillin tosilate hydrate |  | Fosfomycin | Fosfomycin sodium |
|  | Piperacillin sodium |  | Aminoglycosides | Streptomycin sulfate |
|  | Ampicillin sodium, Cloxacillin sodium hydrate |  |  | Kanamycin sulfate |
|  | Ampicillin sodium, Sulbactam sodium |  |  | Gentamicin sulfate |
|  | Amoxicillin hydrate, Clavulanate potassium |  |  | Tobramycin |
|  | Tazobactam, Piperacillin hydrate |  |  | Dibekacin sulfate |
|  |  |  |  | Amikacin sulfate |
| Cephalosporins | Cefalotin sodium |  |  | Isepamicin sulfate |
|  | Cefazolin sodium |  |  | Arbekacin sulfate |
|  | Cefotiam hydrochloride |  | Macrolide | Erythromycin ethylsuccinate |
|  | Cefmetazole sodium |  |  | Erythromycin |
|  | Cefminox sodium hydrate |  |  | Clarithromycin |
|  | Flomoxef sodium |  |  | Roxithromycin |
|  | Cefotaxime sodium |  |  | Azithromycin hydrate |
|  | Cefoperazone sodium |  |  | Josamycin |
|  | Cefoperazone sodium, Sulbactam sodium |  |  | Spiramycin acetate |
|  | Cefmenoxime hemihydrochloride |  | Tetracycline | Tetracycline hydrochloride |
|  | Ceftriaxone sodium |  |  | Demethylchlortetracycline hydrochloride |
|  | Ceftazidime hydrate |  |  | Doxycycline hydrochloride hydrate |
|  | Latamoxef sodium |  |  | Minocycline hydrochloride |
|  | Cefpirome sulfate |  | Lincomycin | Lincomycin hydrochloride hydrate |
|  | Cefozopran hydrochloride |  |  | Clindamycin |
|  | Cefepime hydrochloride hydrate |  | Chloramphenicol | Chloramphenicol sodium succinate |
|  | Cefalexin |  | Fluoroquinolone | Norfloxacin |
|  | Cefalexin Granules |  |  | Ofloxacin |
|  | Cefroxadine hydrate |  |  | Levofloxacin hydrate |
|  | Cefaclor |  |  | Ciprofloxacin hydrochloride |
|  | Cefaclor Granules |  |  | Lomefloxacin hydrochloride |
|  | Cefotiam hexetil hydrochloride |  |  | Tosufloxacin tosilate hydrate |
|  | Cefuroxime axetil |  |  | Pazufloxacin mesilate |
|  | Cefdinir |  |  | Prulifloxacin |
|  | Ceftibuten hydrate |  |  | Moxifloxacin hydrochloride |
|  | Cefditoren pivoxil |  |  | Garenoxacin mesilate hydrate |
|  | Cefixime |  |  | Sitafloxacin hydrate |
|  | Cefteram pivoxil |  | Sulfonamide antibiotic | Sulfamethoxazole/Trimethoprim |
|  | Cefpodoxime proxetil |  |  |  |
|  |  |  | Corticosteroids | Prednisolone |
| Carbapenem | Imipenem/Cilastatin sodium |  |  | Betamethasone |
|  | Panipenem/Betamipron |  |  | Hydrocortisone |
|  | Meropenem hydrate |  |  | Dexamethasone |
|  | Biapenem |  |  | Methylprednisolone |
|  | Doripenem hydrate |  |  | Triamcinolone |
|  | Tebipenem pivoxil |  |  |  |

**Table S3 Detailed age distribution of participants among disaster victims and non-victims**

| Age | Nonvictims | Victims |
| --- | --- | --- |
| 40-44 | 409,441 | 2,030 |
| 45-49 | 412,617 | 1,993 |
| 50-54 | 349,813 | 1,708 |
| 55-59 | 341,148 | 2,010 |
| 60-64 | 357,017 | 2,444 |
| 65-69 | 456,621 | 4,030 |
| 70-74 | 398,373 | 3,963 |
| 75-79 | 328,572 | 3,244 |
| 80- | 612,411 | 5,159 |

**Table S4. Multivariate logistic regression analysis for baseline (before disaster) prescriptions of long-acting inhaled medications for COPD.**

| **Variables** | **Odds ratio  ( 95% confidence Interval)** | **P-value** |
| --- | --- | --- |
| Victims (vs. non-victims) | 1.144 (0.985 – 1.328) | 0.078 |
| Age group (≧65 vs. <65) | 8.994 (8.589 – 9.417) | <0.001 |
| Female (vs.male) | 0.143 (0.137 – 0.149) | <0.001 |

Definition of abbreviations: COPD = chronic obstructive pulmonary disease

**Table S5 New prescription of long-acting inhaled medications for COPD after the 2018 Japan floods, n (%)**

|  | **Victims (n = 26,404)** | **Non-victims (n = 3,648,131)** |
| --- | --- | --- |
| All people, n (%) | 80 (0.35) | 6,164 (0.21) |
| Age group, n (%) |  |  |
| 40-64 years | 13 (0.13) | 1,230 (0.07) |
| ≥ 65 years | 67 (0.41) | 4,934 (0.28) |
| Sex, n (%) |  |  |
| Male, n (%) | 69 (0.60) | 4,978 (0.30) |
| Female, n (%) | 11 (0.07) | 1,186 (0.06) |

Abbreviations: COPD, chronic obstructive pulmonary disease

**Table S6. Difference-in-difference analysis of changes in prescription rates of long-acting inhaled COPD medications between disaster victims and non-victims before and after the flood (n = 3,692,594)**

| Odds Ratio  (95% Confidence Interval) | P-value |
| --- | --- |
| 1.18 (1.06 - 1.32) | 0.004 |

**Figure S1. Subgroup analysis of prescription for long-acting inhaled medications for chronic obstructive pulmonary disease** (A) 65–79 years old, (B) ≥80 years old

A	B

**Figure S2. Monthly time-course of adjusted ratios of odds ratios for prescriptions of exacerbation-related medications in subgroup analyses comparing disaster victims and non-victims.** (A) 65–79 years old, (B) ≥80 years old

A	B

Adjusted ratio of odds ratios

|  | y=1 |
| --- | --- |
| -12 | 1 |
| -11 | 1 |
| -10 | 1 |
| -9 | 1 |
| -8 | 1 |
| -7 | 1 |
| -6 | 1 |
| -5 | 1 |
| -4 | 1 |
| -3 | 1 |
| -2 | 1 |
| -1 | 1 |
| 0 | 1 |
| 1 | 1 |
| 2 | 1 |
| 3 | 1 |
| 4 | 1 |
| 5 | 1 |
| 6 | 1 |
| 7 | 1 |
| 8 | 1 |
| 9 | 1 |
| 10 | 1 |
| 11 | 1 |

| Month | Adjusted ratio of odds ratios |
| --- | --- |
| -11 | 0.99439 |
| -10 | 1.51475 |
| -9 | 1.01276 |
| -8 | 1.45333 |
| -7 | 1.0966 |
| -6 | 1.26399 |
| -5 | 1.03844 |
| -4 | 1.22312 |
| -3 | 0.971498 |
| -2 | 1.1937 |
| -1 | 0.649103 |
| 0 | 0.999532 |
| 1 | 1.27777 |
| 2 | 1.17268 |
| 3 | 1.57858 |
| 4 | 1.15727 |
| 5 | 1.53018 |
| 6 | 1.09476 |
| 7 | 1.01581 |
| 8 | 1.12818 |
| 9 | 1.16688 |
| 10 | 1.01261 |
| 11 | 1.15736 |

| Month | x=0 |
| --- | --- |
| 0 | 4 |
| 0 | 0 |

Adjusted ratio of odds ratios

|  | y=1 |
| --- | --- |
| -12 | 1 |
| -11 | 1 |
| -10 | 1 |
| -9 | 1 |
| -8 | 1 |
| -7 | 1 |
| -6 | 1 |
| -5 | 1 |
| -4 | 1 |
| -3 | 1 |
| -2 | 1 |
| -1 | 1 |
| 0 | 1 |
| 1 | 1 |
| 2 | 1 |
| 3 | 1 |
| 4 | 1 |
| 5 | 1 |
| 6 | 1 |
| 7 | 1 |
| 8 | 1 |
| 9 | 1 |
| 10 | 1 |
| 11 | 1 |

| Month | Adjusted ratio of odds ratios |
| --- | --- |
| -11 | 0.830611 |
| -10 | 1.22791 |
| -9 | 0.770753 |
| -8 | 1.22681 |
| -7 | 0.924233 |
| -6 | 1.04509 |
| -5 | 1.1931 |
| -4 | 1.22567 |
| -3 | 1.28699 |
| -2 | 1.36356 |
| -1 | 0.793527 |
| 0 | 1.52477 |
| 1 | 1.72644 |
| 2 | 1.40758 |
| 3 | 1.87906 |
| 4 | 1.72886 |
| 5 | 2.27097 |
| 6 | 1.90725 |
| 7 | 1.16638 |
| 8 | 1.54952 |
| 9 | 1.50171 |
| 10 | 1.82529 |
| 11 | 1.36681 |

| Month | x=0 |
| --- | --- |
| 0 | 6 |
| 0 | 0 |
